# Aetiologies of bacterial tick-borne febrile illnesses in humans in Africa: diagnostic limitations and the need for improvement

**DOI:** 10.3389/fmed.2024.1419575

**Published:** 2024-09-16

**Authors:** Abdulrahman Adamu, Flavia Reyer, Nafiú Lawal, Abdurrahman Jibril Hassan, Mustapha Umar Imam, Muhammad Bashir Bello, Peter Kraiczy

**Affiliations:** ^1^Centre for Advanced Medical Research and Training, Usmanu Danfodiyo University Sokoto, Sokoto, Nigeria; ^2^Department of Animal Health and Production Technology, Federal Polytechnic Bali, Taraba State, Nigeria; ^3^Department of Veterinary Microbiology, Usmanu Danfodiyo University Sokoto, Sokoto, Nigeria; ^4^Goethe University Frankfurt, Institute of Medical Microbiology and Infection Control, University Hospital of Frankfurt, Frankfurt, Germany; ^5^Department of Veterinary Public and Preventive Medicine, Usmanu Danfodiyo University Sokoto, Sokoto, Nigeria; ^6^Department of Medical Biochemistry, Usmanu Danfodiyo University Sokoto, Sokoto, Nigeria; ^7^Infectious Disease Research Department, King Abdullah International Medical Research Center, Riyadh, Saudi Arabia

**Keywords:** Africa, zoonoses, tick-borne disease, aetiologies, human health, *in vitro* diagnostics

## Abstract

Tick-borne febrile illnesses caused by pathogens like *Anaplasma* spp., *Bartonella* spp., *Borrelia* spp., *Ehrlichia* spp., *Coxiella burnetii*, *Francisella tularensis*, and *Rickettsia* spp., are significant health concerns in Africa. The epidemiological occurrence of these pathogens is closely linked to the habitats of their vectors, prevalent in rural and semi-urban areas where humans and livestock coexist. The overlapping clinical presentations, non-specific symptoms, and limited access to commercially available *in vitro* diagnostics in resource-limited settings exacerbate the complexity of accurate diagnoses. This review aimed to systematically extract and analyze existing literature on tick-borne febrile illnesses in Africa, highlighting the diagnostic challenges and presenting an up-to-date overview of the most relevant pathogens affecting human populations. A comprehensive literature search from January 1990 to June 2024 using databases like PubMed, Cochrane Library, Science Direct, EMBASE, and Google Scholar yielded 13,420 articles, of which 70 met the inclusion criteria. *Anaplasma* spp. were reported in Morocco, Egypt, and South Africa; *Francisella* spp. in Kenya and Ethiopia; *Ehrlichia* spp. in Cameroon; *Bartonella* spp. in Senegal, Namibia, South Africa, and Ethiopia; *Borrelia* spp. in Senegal, Gabon, Tanzania, and Ethiopia; *Coxiella burnetii* in 10 countries including Senegal, Mali, and South Africa; and *Rickettsia* spp. in 14 countries including Senegal, Algeria, and Uganda. Data were analyzed using a fixed-effect model in R version 4.0.1 and visualized on an African map using Tableau version 2022.2. This review highlights the urgent need for improved diagnostics to better manage and control tick-borne febrile illnesses in Africa.

## 1 Introduction

**Figure 1:**
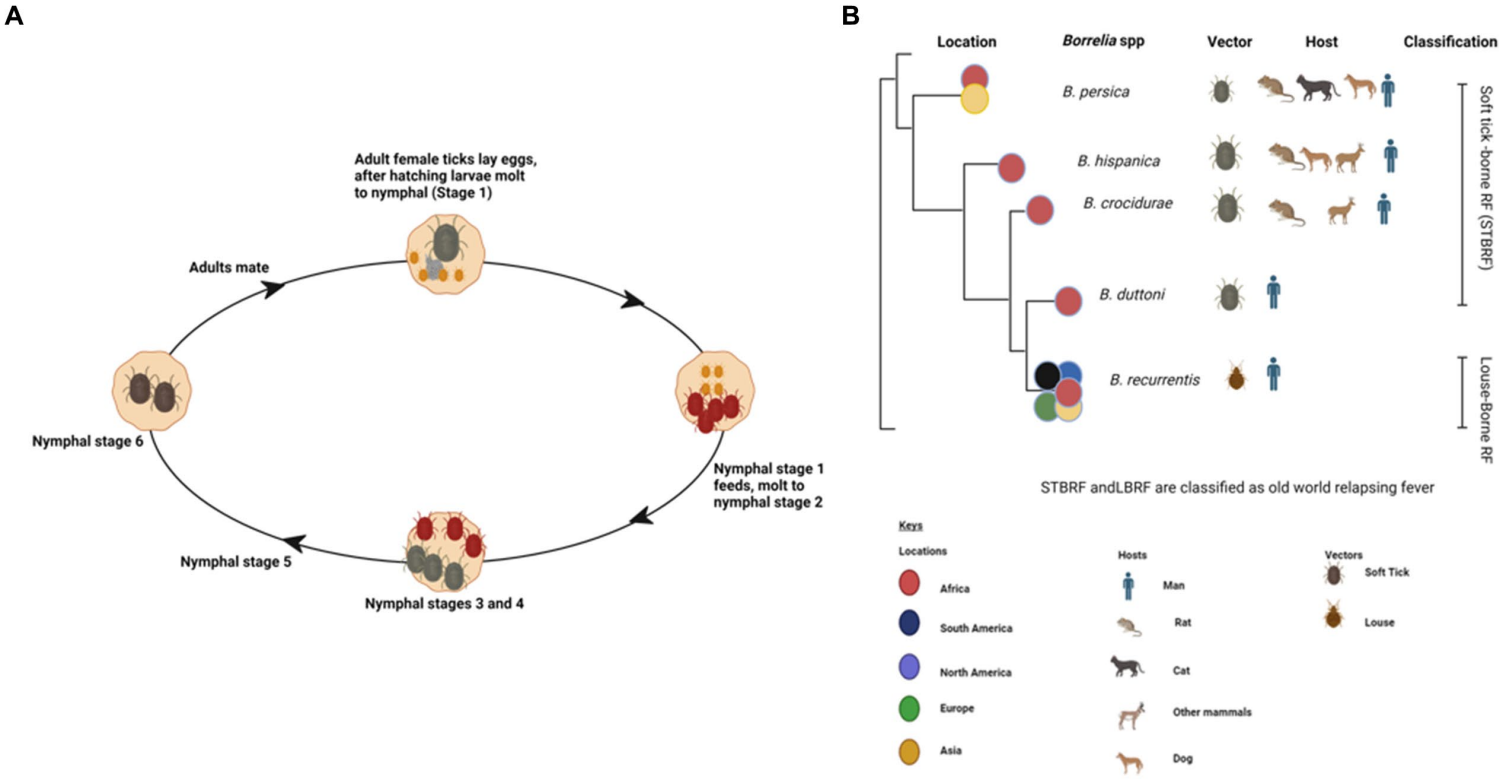
Various life stages of a soft tick (*Ornithodoros spp.*) created with BioRender **(A)**. Phylodendrogram of average nucleotide identity values among RF borreliae genomes copied and modified from (16) with BioRender, licensed under CC BY. **(B)**.

Ticks are parasitic arachnids that feed on the blood of different hosts including reptiles, birds, and mammals (1). They can be categorized as hard ticks belonging to the suborder *Ixodidae* or soft ticks within the suborder *Argasidae* (2). Soft ticks are usually distinguished by their distinctive fast feeding, which lasts approximately 20–30 min. In contrast to ixodid ticks, soft ticks can undergo multiple nymphal stages (Figure 1A) (3). The prerequisite for molting typically involves the consumption of a blood meal from a vertebrate host (4). However, certain tick species might undergo multiple feedings before progressing to the next developmental stage (1, 2). Soft ticks exhibit remarkable longevity as exemplified by *Ornithodoros turicata* (3). Worldwide, more than 800 tick species have been identified, which are known to transmit a variety of diverse viruses, bacteria, and protozoans (5).

Ticks are the most significant transmitting vectors of human pathogens after mosquitoes, posing a substantial threat to both human and veterinary health (6). Globally, both vector-borne diseases make substantial contributions to Acute Febrile Illness (AFI) (7). However, Africa is considered to be a hotspot for a multitude of tick-borne pathogens. Recently, the significance of tick-borne diseases has been emphasized, particularly concerning their impact on the well-being of economically disadvantaged farming communities in developing countries (8, 9). In sub-Saharan Africa, there is a significant lack of access to reliable *in vitro* diagnostics, leading to frequent misdiagnoses, even though an accurate diagnosis is crucial for disease prevention and treatment (10). Currently, the gold standard for diagnosing tick-borne diseases still involves microscopic visualization of Giemsa-stained thick blood smears, known to lack sensitivity and specificity for discriminating the causative pathogen (11). A novel approach for the serodiagnosis of louse-borne relapsing fever with high sensitivity and specificity for both IgM and IgG has recently been developed (12). Alternatively, for the identification of tick species and transmitted pathogens more sensitive methods like DNA amplification techniques are being developed (13). However, the application of molecular techniques is constrained by the necessity for specialized and cost-intensive equipment and well-trained technicians. This review explores the aetiologies of tick-borne febrile illnesses in Africa, providing insight into the diverse causative agents, their epidemiological patterns, geographical distribution, and the challenges faced in distinguishing between these etiological agents.

## 2 Methodology

**Figure 2:**
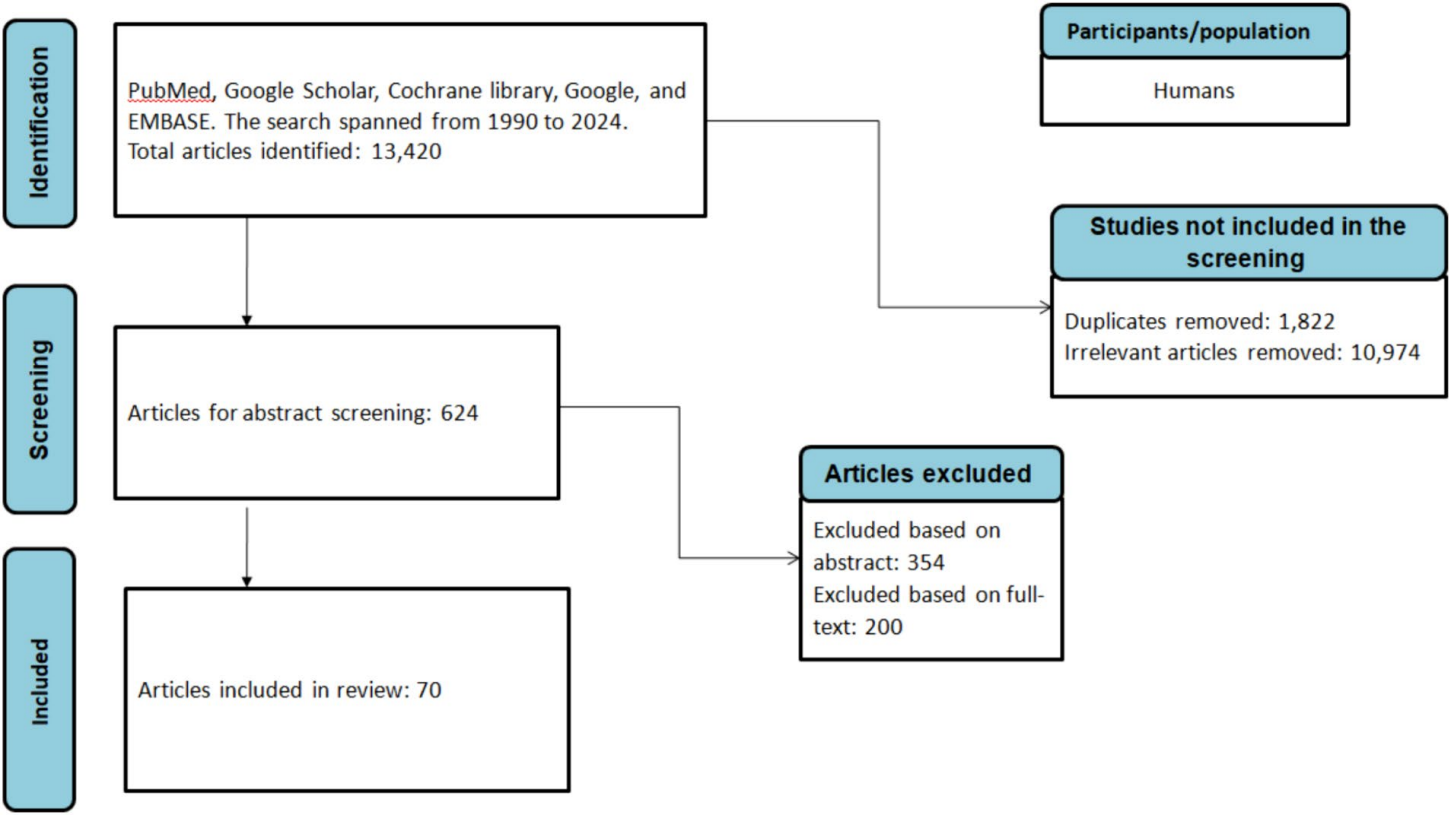
Flowchart of the literature selection process.

A comprehensive literature search was conducted using the following databases: PubMed, Cochrane Library, Science Direct, EMBASE, and the search engine Google Scholar. The search spanned from January 1990 to June 2024 and utilized keywords such as “Anaplasmosis,” “Borreliosis,” “Bartonellosis,” “Q-Fever,” “*Coxiella burnetii,” “Borrelia,” “Bartonella,” “Ehrlichia,”* “Ehrlichiosis,” “*Francisella tularensis*,” “Rickettsiosis,” “Tick-borne relapsing fever,” combined with “Africa,” “African countries,” and individual country names within Africa (Figure 2).

### 2.1 Inclusion criteria

Only studies involving humans infected with *Anaplasma phagocytophilum, Bartonella* spp.*, Borrelia* spp.*, Coxiella burnetii* (Q-fever), *Ehrlichia* spp.*, Francisella tularensis*, and *Rickettsia* spp. Observational studies, including cross-sectional, prevalence, case–control, and cohort studies were included Studies published in English.

### 2.2 Exclusion criteria

Studies involving animals and ticks without a focus on human infections. Similarly, short reports, incomplete articles, and review articles were excluded.

## 3 Ticks as vectors of emerging and re-emerging human diseases

**Figure 3:**
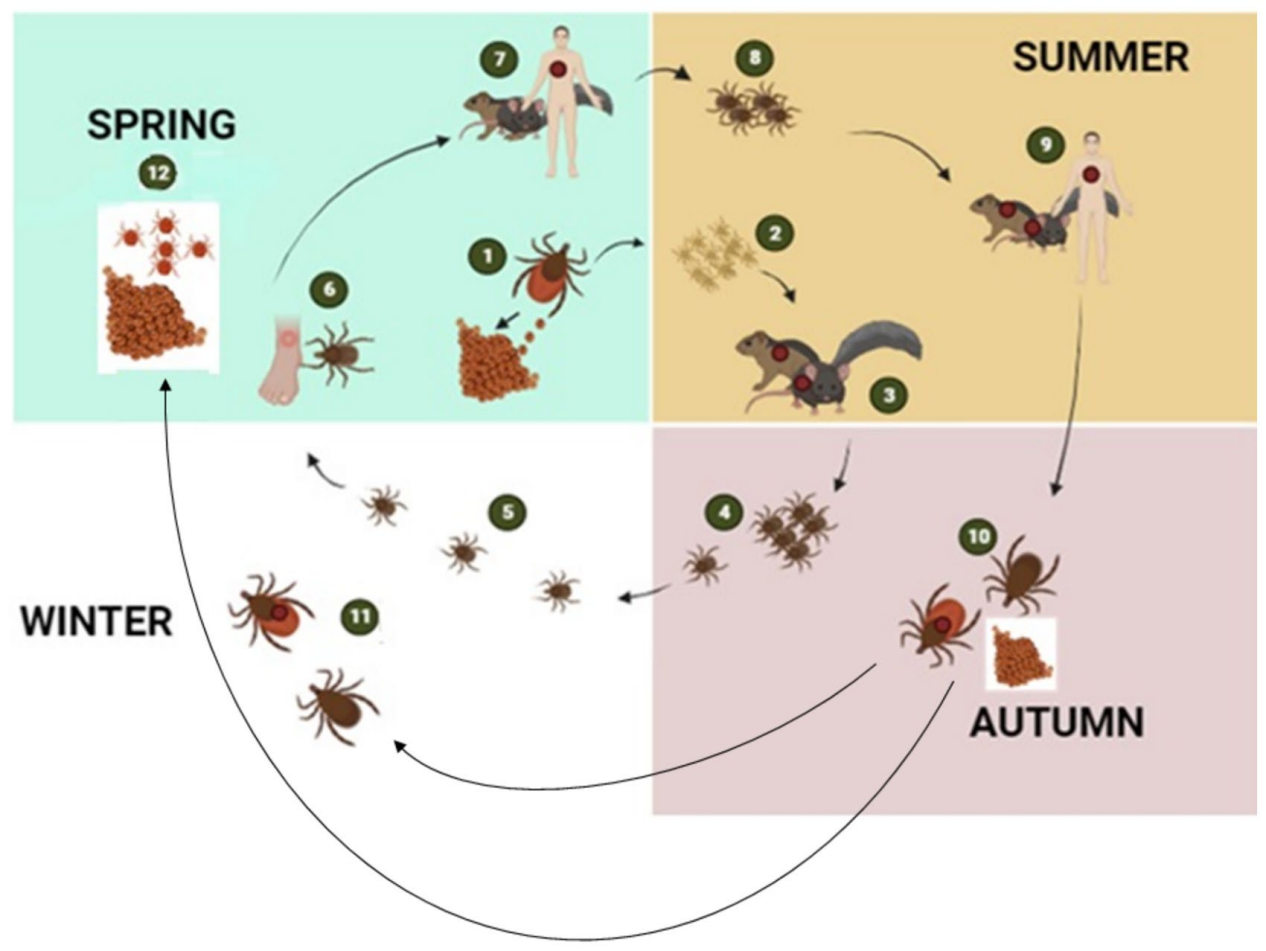
The figure illustrates the transmission cycle of hard ticks through different seasons of the year: The tick life cycle begins with female ticks laying eggs in the spring (1), which hatch in spring or early summer giving rise to larvae (2). During late summer, larvae become infected with bacterial pathogens when they feed on infected mice or other small rodent hosts (3). The larvae then moult into nymphs (4) and overwinter in the environment (5). By the following spring, questing nymphs again feed either on humans (6) or on wild animals (7). After feeding, nymphs drop off the host to molt into adults (8). Adult ticks then continue to feed on larger mammalian hosts including humans and diverse mammalian hosts (9). Engorged female ticks drop off the host to lay eggs and then died (10). Adult ticks remain dormant in the environment during winter when they did not feed on a host (11). With the onset of spring, the cycle repeats by the next generation of larvae (12) (2, 5) created with BioRender.com.

Ticks, belonging to the arachnid class, are ectoparasites that feed on blood from reptiles, birds, and mammals. They include three well-established families: *Ixodidae* or “hard ticks” (694 species), *Argasidae* or “soft ticks” (177 species), and *Nuttalliellidae* represented by a single species confined to southern Africa (1, 2, 14). The *Ixodidae* family is characterized by a three-host life cycle and features four distinct stages: egg, larva, nymph, and adult. Each of these stages typically parasitizes a different host (Figure 3). Prior to feeding, ixodid ticks attach to their hosts, which are often small mammals, for several hours. During the feeding process, ticks can acquire pathogens from the infected hosts (4, 15). In contrast to soft ticks, hard ticks feed only once on a mammalian host, most often small rodents and birds but also humans, before progressing to the next developmental stage, as depicted in Figure 3. Soft ticks have a multi-host life cycle (Figure 1A) (16) that can involve multiple feedings at each stage, but they do not spend extended periods attached to their hosts (Figure 1B). Instead, they feed quickly, often within a matter of minutes to an hour. One remarkable feature of soft ticks is their longevity; for example, *Ornithodoros turicata* can survive for up to 7 years without feeding and nearly 10 years with periodic blood meals (3). Soft ticks tend to live in close proximity to their hosts, such as in burrows or nests, allowing them to feed repeatedly on the same hosts over time. The transmission of pathogens from one generation of ticks to the next through transovarial means introduces a significant level of complexity to the factors influencing disease maintenance and spread (Figure 1A) (17). This transovarial transmission is observed in both hard and soft ticks, although it is more common and well-studied in hard ticks. Soft ticks, however, can also transmit pathogens through their brief yet repeated feeding sessions, increasing the risk of pathogen spread (17).

## 4 Pathogens responsible for human tick-borne bacterial febrile illnesses in Africa

### 4.1 Anaplasmosis

Human Anaplasmosis is primarily caused by *Anaplasma phagocytophilum* and *A. platys*, both are obligate intracellular Gram-negative bacteria (18). Various possibilities of transmission have been documented, including nosocomial infection by direct contact with blood and respiratory secretions, as well as transmission through blood transfusions (19, 20). *Anaplasma phagocytophilum* is primarily transmitted by various hard tick species within the *Ixodes* genus (21, 22). While *Amblyomma*, *Dermacentor*, *Hyalomma*, and other ixodid ticks could potentially contribute to the transmission cycle of this bacterium in Africa (23, 24). The prevalence and distribution of these ticks vary, and there are regional differences in the tick species associated with *A. phagocytophilum* transmission. However, their role as vectors remains uncertain (23).Human anaplasmosis manifests as a febrile illness and is characterized by symptoms such as fever, headache, muscle aches, and fatigue. The disease primarily affects white blood cells, leading to a reduction in their numbers and impacting the immune response (25).

**Figure 4:**
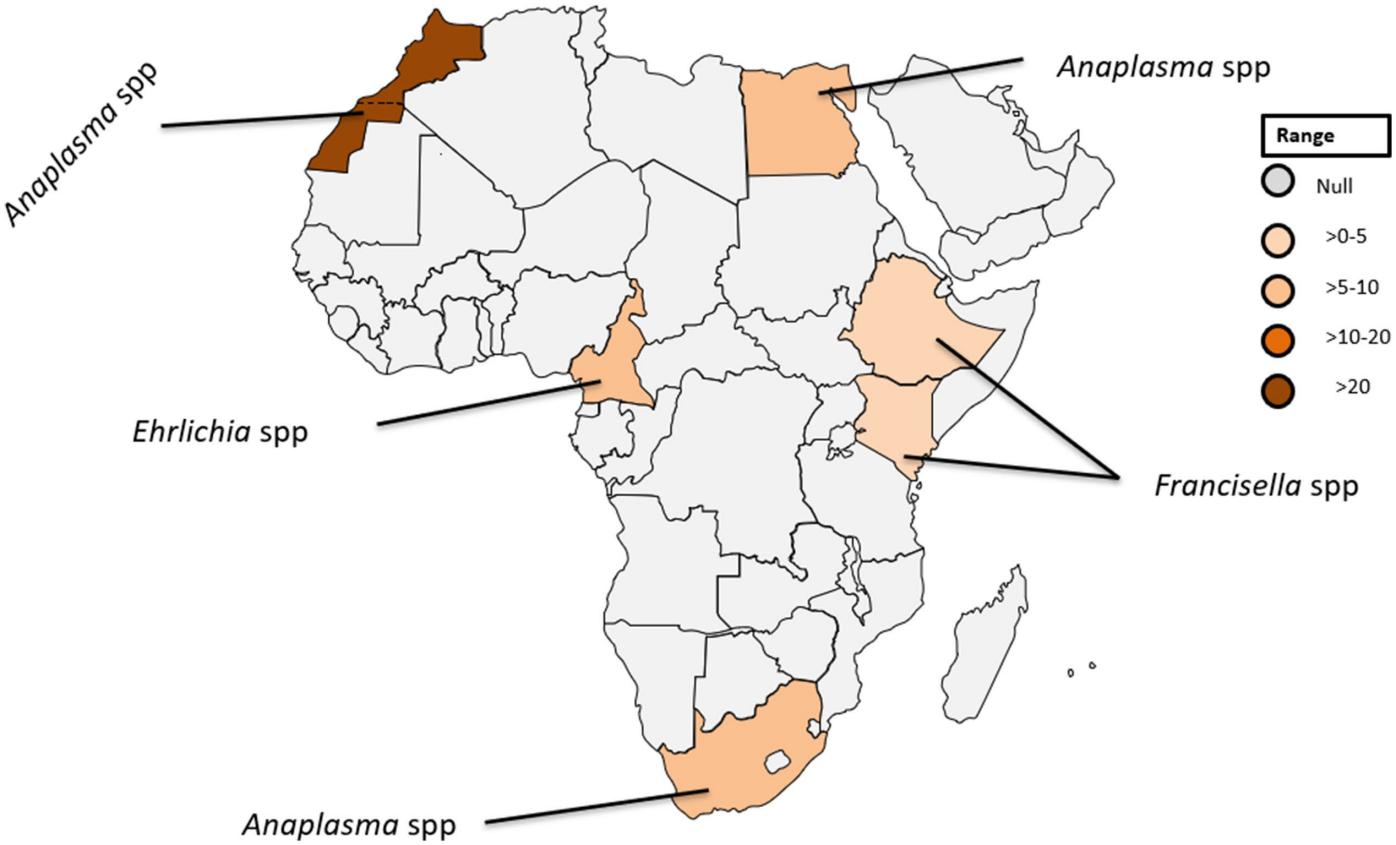
Pooled data using fixed effect model with R version 4.0.1 showing reported Prevalence of *Anaplasma* spp. *Ehrlichia* spp. and *Francisella tularensis* in different African countries designed using Tableau, 2022.2 software.

In the African context, *A. phagocytophilum* has only been identified in soft ticks collected in temperate North Africa (26). In Egypt, infections with *A. phagocytophilum* have been reported in five individuals, accounting for 7.5% of the studied population (see Table 1; Figure 4), highlighting the significance of understanding the prevalence of tick-borne pathogens in the context of the One Health concept (27).

**Table 1:** Prevalence of bacterial species detected in humans in different countries of Africa.

Bacteria species	Research period	Country	Total sample tested	No of positive cases	% of positive cases	Diagnostic method	Sample tested	Genes/Proteins	Reference
*Anaplasma phagocytophilum*	2005	Egypt	67	5	7.5	PCR	Blood	16S rRNA	(27)
	2015	Morocco	253	92	36.4	IFA	Blood	n.a.	(28)
	2013–2015	Morocco	10	7	70	ELISA	Serum	n.a.	(115)
	2013–2015	South Africa	74	4	5.4	PCR	Blood	16S rRNA	(116)
	2015	Morocco	115	25	21.7	ELISA	Serum	n.a.	(28)
*Bartonella* spp.	2013	Ethiopia	394	1	0.01	PCR	Whole blood	16S rRNA	(14)
	2019–2020	South Africa	14	6	43	PCR	Blood	16S rRNA	(50)
	2006–2015	Ethiopia	574	5	0.9	PCR	Blood	n.a.	(51)
	2011–2012	Namibia	105	3	2.9	ELISA (IgG)	Serum	n.a.	(52)
	2017	South Africa	74	7	9.5	PCR	Blood	n.a.	(94)
	2011–2012	Senegal	440	23	4.3	POC-qPCR	Blood drops (3–4)	16S-23S internal transcribed spacer (ITS2)	(105)
*Borrelia* spp.	2008–2009	Senegal	206	27	13	GS (microscopy)	Whole blood	16S rRNA	(107)
	2008–2009	Senegal	20	2	0.3	qPCR	Whole blood	16S rRNA	(107)
	2013	Ethiopia	394	3	0.8	PCR	Whole blood	16S rRNA	(14)
	2009–2010	Ethiopia	102	2	2	qPCR	n.a.	16S rRNA	(96)
	2011–2012	Gabon	100	2	2	PCR	Blood	ITS4, *flaB*	(72)
	2011–2012	Senegal	440	35	9.5	POC-q PCR	Blood drops (3–4)	16S rRNA	(105)
	2005	Egypt	67	2	3	PCR	Blood	16S rRNA	(27)
	2003	Tanzania	54	6	11	PCR	Blood	Flagellin	(117)
	2003	Tanzania	307	13	4	PCR	Blood	Flagellin	(117)
	2010–2012	Gabon	100	2	2	PCR	Blood	ITS4	(72)
	1990	Senegal	1,340	12	0.9	Blood smear	Blood	n.a.	(118)
	2016	Senegal	800	94	12	PCR	Blood	*16S* rRNA	(119)
	1990–2003	Senegal	4,599	800	17.4	PCR	Blood	*n.a.*	(70)
	2018–2019	Senegal	213	33	15.5	PCR	Blood	*16S rRNA*	(120)
	2012	Ethiopia	407	10	2.5	Microscope	Blood	*n.a.*	(121)
	2022	Ethiopia	36	14	38.9	Microscope	Blood film	*n.a.*	(122)
*Coxiella burnetii*	2012–2016	South Africa	139	37	27	ELISA	Blood	n.a.	(93)
	2012–2013	South Sudan	632	12	2	ELISA	Blood	n.a.	(80)
	2017	South Africa	73	28	38.3	PCR	Blood	n.a.	(94)
	2017	South Africa	138	n.a.	12.3	ELISA	n.a.	n.a.	(94)
	2011–2012	Namibia	276	72	26.1	IFA and ELISA (IgG)	Serum	n.a.	(52)
	2007–2008	Tanzania	870	483	55.5	ELISA, IFA	Blood specimens	n.a.	(123)
	2004	Tunisia	47	4	8.5	IFA	Blood	n.a.	(103)
	2016	Sao Tome	240	16	6.7	ELISA	Blood	n.a.	(100)
	2014	Gambia	599	23	3.8	ELISA	Blood	n.a.	(79)
	2011–2012	Senegal	440	2	0.5	POC-qPCR	Blood drops (3–4)	Spacer IS1111	(105)
	2008	Gambia	796	66	8.3	ELISA	Blood	n.a.	(124)
	1999	Zambia	377	31	8.3	ELISA	Blood	n.a.	(125)
	2016	Kenya	2,049	52	2.5	ELISA	Blood	n.a.	(126)
	2002–2003	Mali	156	63	40.4	ELISA	Blood	n.a.	(127)
	1994	Niger	177	17	9.6	ELISA	Blood	n.a.	(128)
	1993	Tunisia	500	130	26	IFA	Blood	n.a.	(129)
*Ehrlichia* spp.	2003	Cameroun	118	12	10	qPCR	Blood	*dsb*	(35)
*Francisella* spp.	2013	Ethiopia	394	3	0.8	PCR	Whole blood	*lpnA*	(14)
	2014–2015	Kenya	730	27	3.7	ELISA	Serum	n.a	(130)
*Rickettsia* spp.	2010	Senegal	238	51	21.4	IFA	Whole blood	n.a.	(87)
	2014	Senegal	68	5	7.4	qPCR	Eschars	*gltA*, *orfB*	(117)
	2015	Tanzania	149	6	4	RT-PCR	Blood	n.a.	(88)
	2010	Cameroun	903	243	26.9	IFA	Blood	n.a.	(89)
	2015–2016	Gabon	428	4	0.9	qPCR, PCR	Blood	*β-actin*	(131)
	2011–2012	Gabon	893	10	1.1	PCR	Blood	*β-actin*	(72)
	2012–2016	South Africa	118	24	21	IFA	Blood	n.a.	(93)
	2012–2013	Sudan	632	25	4	ELISA	Blood	n.a.	(80)
	2017	South Africa	71	45	63.4	PCR	Blood	n.a.	(94)
	2017	South Africa	138	33	24.1	ELISA	n.a.	n.a.	(94)
	2008–2010	Senegal	451	20	4.4	qPCR	Fecal	*gltA*	(132)
	2008–2010	Senegal	230	18	7.8	qPCR	Fecal	*gltA*	(132)
	2009–2010	Ethiopia	102	3	2.9	qPCR	n.a.	*gltA*	(96)
	2011–2012	Namibia	269	32	11.9	ELISA (IgM)	Serum	n.a.	(52)
	2011–2012	Namibia	269	40	14.9	ELISA (IgG)	Serum	n.a.	(52)
	2013–2015	Algeria	166	9	14.7	qPCR, IFA	Sera	*gltA*	(133)
	2007–2008	Tanzania	1,228	884	67.9	IFA	Serum	n.a.	(98)
	2007–2008	Tanzania	870	450	51.7	IFA	Blood specimens	n.a.	(123)
	2004–2005	Algeria	248	191	77	IFA	Serum	n.a.	(99)
	2004–2005	Algeria	44	29	61.4	PCR	Eschar	*ompA*	(99)
	2010	Djibouti	49	8	16	ELISA	Blood	n.a.	(101)
	2004	Tunisia	47	31	57.5	IFA	Blood	n.a.	(103)
	2010	Madagascar	1,229	20	1.63	IFAT	Plasma	n.a.	(97)
	2007–2012	Tunisia	180	82	57.7	PCR	Blood	n.a.	(134)
	2016	Sao Tome	240	20	8.3	ELISA	Blood	n.a.	(100)
	2012–2014	Tunisia	121	56	46.2	qPCR	Blood sample	n.a.	(104)
	2012–2014	Tunisia	121	35	28.9	RLB	Blood sample	n.a.	(104)
	2011–2013	Uganda	1,281	97	7.6	IFA	Blood	n.a.	(90)
	2010–2012	Gabon	793	8	1	PCR	Blood	n.a.	(72)
	2013	Ethiopia	394	3	0.8	PCR	Blood	23S-5S rRNA	(14)
	2024	Senegal	213	8	3.8	PCR	Blood	16S	(120)

CSF, cerebrospinal fluid; *dsbA*, thio-disulfideoxidoreductase A; *gltA*, citrate synthase; GS, Giemsa stain of thin and thick blood smears; IFA, immunofluorescence assay; *lpnA*, lipoprotein A; MRI: magnetic resonance imaging; *ompA*, outer membrane protein A; *orf*, open reading frame; RLB, reverse line blot, POC-qPCR; Point of Care-qPCR; IFAT, indirect fluorescent antibody test; n.a., not available.

An investigation dealing with the prevalence and risk factors of *A. phagocytophilum* exposure in Morocco, including the analysis of seropositivity rates among dog handlers and blood donors, disclosed rates of 37 and 27% among dog handlers and 36 and 22% among blood donors, respectively. This revealed no statistically significant differences between the two groups, underscoring the overall frequent incidence of *A. phagocytophilum* exposure in both, high-risk populations and blood donors in Morocco (28).

The diagnosis of human anaplasmosis in various African countries has not received sufficient attention in recent years, as no reported studies are found in the selected databases since 2000. This obvious gap of knowledge highlights the need for further investigations aiming to collect data on the prevalence and impact of *Anaplasma* infections in different countries of Africa. Notably, North Africa has emerged as a focus of research on *Anaplasma* infections where *A. phagocytophilum* was initially identified in human body lice obtained from homeless individuals in three distinct cities in northern Algeria. The prevalence of *A. phagocytophilum* in these arthropods was recorded at 0.76% (29), highlighting the role of body lice as potential vectors for *Anaplasma* transmission and raising awareness among public health authorities.

Furthermore, intriguingly, a potentially novel species of *Anaplasma* spp. was identified in head lice collected from patients in Mali, with a prevalence of 0.3%, as described by Amanzougaghene et al. (30). Emphasizing the diverse nature of *Anaplasma* species and the importance of exploring the vector competence of different arthropods will increase the understanding of the transmission dynamics of these particular pathogens.

### 4.2 Ehrlichiosis

*Ehrlichiae* are obligate intracellular Gram-negative bacteria belonging to the same family as *Anaplasma*, exhibiting a predilection for monocytes and granulocytes similar to *Anaplasma* spp. (31). The prevalence of these human pathogenic microorganisms is widespread worldwide, and their presence significantly impacts the occurrence of ehrlichiosis in both humans and animals (29).

Within the genus *Ehrlichia*, *Ehrlichia (E.) chaffeensis*, and *E. ewingii* stand out as causative agents of ehrlichiosis in humans (30, 32). *E. chaffeensis*, responsible for human monocytic ehrlichiosis was initially identified in the United States (33). Although there has been a paucity of reported human cases in Africa (34). Remarkably, a singular case was documented in Cameroon where blood samples from 10% of the 118 investigated patients were tested positive for gene-specific *E. chaffeensis* DNA (35). This finding is noteworthy because the sequence obtained exhibit 100% genetic identity among the positive cases to a strain of *E. chaffeensis* isolated in Arkansas, USA (36). The detection of *E. chaffeensis* in Cameroon underscores the global distribution of these pathogens and raises questions about the potential factors influencing their epidemiology in diverse geographic regions. Furthermore, the identification of *E. chaffeensis* in Cameroon serves as a crucial piece of evidence, shedding light on the presence of tick-borne transmitted human pathogens in sub-Saharan Africa (see Table 1; Figure 4).

Due to the scarcity of reports since 2000, additional research is necessary to thoroughly comprehend the prevalence of potential vectors and the biological factors that influence the epidemiology of Ehrlichiae in Africa. While the genus *Ehrlichia* includes several species known to cause diseases in animals and humans, research and literature on *E. ewingii* within the African continent are notably scarce (6). The majority of studies and reports related to *Ehrlichia* species in Africa have primarily focused on *E. chaffeensis* and *E. canis* (6, 35, 37). *E. ewingii* is recognized for its ability to infect both animals and humans, causing a febrile illness known as human granulocytic ehrlichiosis (HGE) (38). The primary vector responsible for the transmission of *E. ewingii* is *Amblyomma americanum* (39). However, there is currently no conclusive evidence confirming the presence of *A. americanum*, and consequently *E. ewingii*, in Africa.

### 4.3 *Francisella* infection

*Francisella*, a genus of gram-negative bacteria, has emerged as a significant pathogen with *Francisella tularensis*, known for causing tularaemia (40). This zoonotic pathogen is highly infectious and primarily transmitted to humans through contact with infected animals, inhalation of contaminated aerosols, ingestion of contaminated water or food, and bites from infected arthropods (41, 42). Tularemia manifests in various types, including the ulceroglandular, glandular, oculoglandular, oropharyngeal, pneumonic, and typhoidal form (43). The clinical presentation can range from mild flu-like symptoms to severe systemic manifestations posing a diagnostic challenge due to its diverse symptoms (43). While historically associated with temperate regions, the prevalence of tularemia in tropical countries is an emerging concern (44). The epidemiology is influenced by factors such as climate, ecosystem dynamics, and interactions between humans and animals (45, 46). *Francisella* spp. exhibits a broad global distribution, with *F. tularensis* subspecies distributed across North America, Europe, Asia, and Africa (40–42). In tropical countries, the prevalence may be influenced by ecological factors including the presence of suitable reservoir hosts and vectors (40). In a recent study conducted in Ethiopia, three out of 394 febrile patients were diagnosed with *Francisella* infection (Figure 4) (14). Diagnosing this infection poses challenges due to the diverse clinical manifestations and the need for specialized laboratory techniques. Serological tests, polymerase chain reaction (PCR), and culture-based methods are commonly employed (15, 47). However, the availability and accessibility of these diagnostic tools in tropical settings may be limited, hindering timely and accurate identification of *Francisella* infections.

### 4.4 Bartonellosis

*Bartonella* spp. are fastidious Gram-negative bacteria responsible for a variety of clinical symptoms summarized as bartonellosis (48). Various *Bartonella* species have been associated with emerging and re-emerging human diseases (49). Throughout history, *Bartonella* spp., including *B. bacilliformis*, *B. quintana*, and *B. henselae*, have been recognized as significant contributors to human disease. While these bacteria are known to cause a range of infections, from mild symptoms such as fever, headache, and malaise to more severe conditions like endocarditis and hallucinations, it’s important to note that they are not the sole causative agents of these diseases (48, 49). Bartonellosis implicated as the cause of blood culture-negative endocarditis (BCNE) is discussed in a previous study conducted in South Africa (50). Similarly, Tasher et al. (51) reported *B. quintana* endocarditis as a rare occurrence in children. The study described five patients from Ethiopia with heart defects and endocarditis caused by either *B. quintana* or an undetermined *Bartonella* species. All patients were afebrile and oligosymptomatic, with three experiencing heart failure. The diagnosis was confirmed by echocardiography, high *Bartonella* IgG titers, and identification of *B. quintana* DNA in the blood sample. The data suggested that *B. quintana* is not uncommon in children with heart defects in Ethiopia and should be considered in cases of culture-negative endocarditis (51).

**Figure 5:**
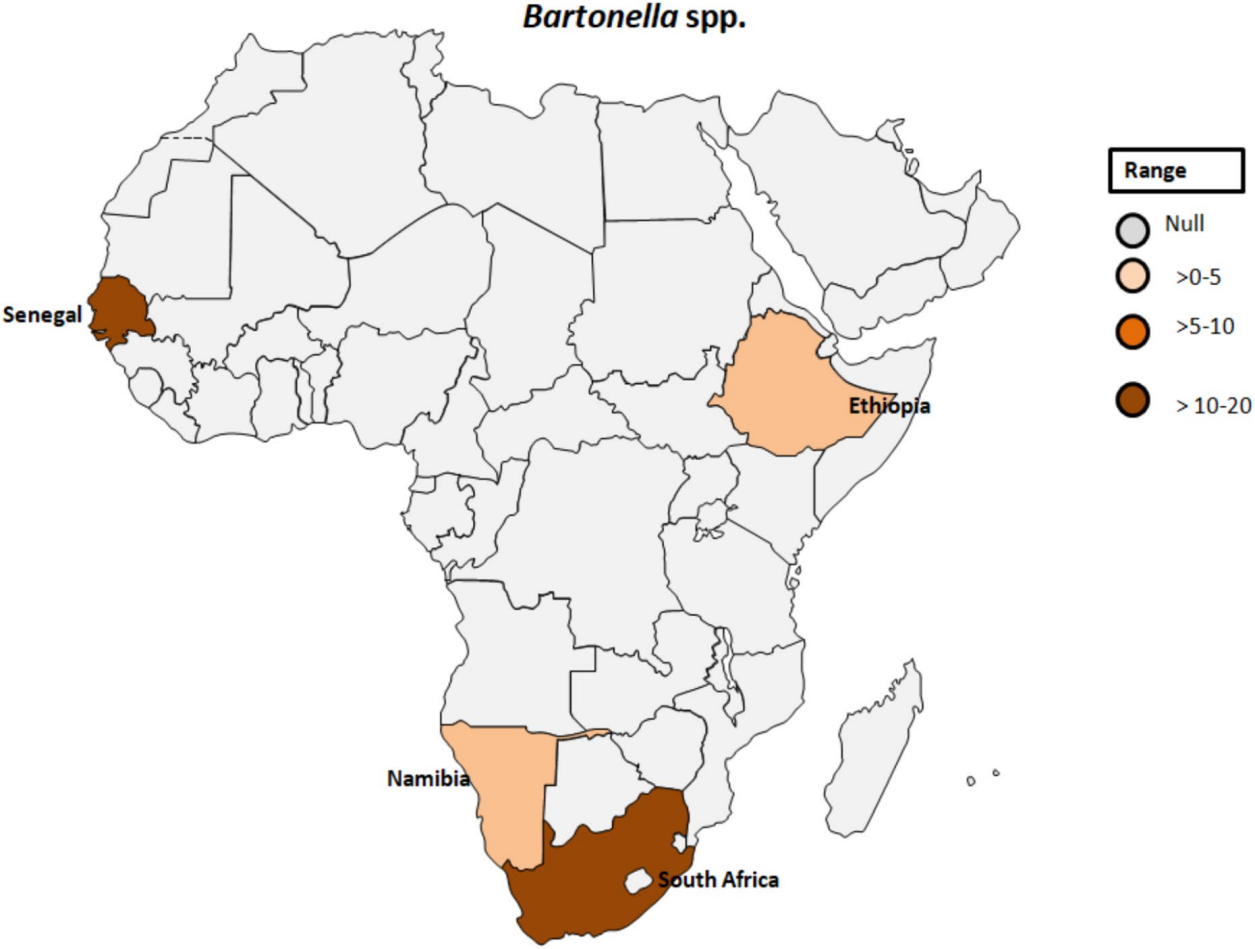
Pooled data using fixed effect model with R version 4.0.1 showing reported Prevalence of *Bartonella* spp. in different African countries designed using Tableau, 2022.2 software.

A study by Noden et al. (52) investigated factors related to exposure to *B. henselae*, revealing a correlation between residing in villages and close association with dogs and cats. Among the 105 samples analyzed for suspected infection with *B. henselae*, three samples (2.9%) tested positive (see Table 1; Figure 5). Additionally, an individual positive for *B. henselae* antibodies also exhibited positivity for *C. burnetii* Phase II antibodies. This finding raises the possibility of coinfections with certain human pathogens circulating in the same area or cross-reactivity, as previously noted (53). The complex interplay between *Bartonella* and other pathogens warrants further investigation to elucidate potential synergies or interactions in environments where the same vectors circulate.

Typically, each *Bartonella* species establishes a specific association with its host, leading to sustained intraerythrocytic bacteremia in the reservoir host, often without detectable harm to the respective host (6, 54). While numerous hematophagous ectoparasites are known to be vectors of *Bartonella*, *ixodid* ticks have also been controversially discussed as potential vectors for *Bartonella* spp. (35).

Arthropods are primary vectors in the spread of *Bartonella* species among mammals including humans, while fleas play a significant role for their distribution (55). Additionally, ticks have been identified as vectors for certain *Bartonella* strains, highlighting their involvement in the transmission cycle (56, 57). Moreover, previous studies suggest that lice may also serve as vectors, emphasizing the adaptability of *Bartonella* to different ectoparasites (58). Notably, the potential for direct transmission from animals to humans through scratches or bites poses another avenue for the spread of *Bartonella* (49, 59). Overall, arthropodsare crucial vectors for unravelling the epidemiology of *Bartonella*-related illnesses and devising effective prevention and control strategies.

### 4.5 Tick-borne relapsing fever

Tick-borne relapsing fever (TBRF) is a vector-borne disease caused by spirochetes belonging to the genus *Borrelia* (2, 60) including *B. duttonii, B. crocidurae, B. hispanica, B. persica*, and *Candidatus Borrelia* kalaharica in Africa and Near East. It is typically transmitted to humans through a bite of infected ticks (Figure 1B) (61–63). In contrast, *B. recurrentis*, the causative agent of louse-borne relapsing fever (LBRF), is the only *Borrelia* spp. vectored by lice (Figure 1B) (60–62, 64). TBRF and LBRF are characterized by recurrent episodes of fever, chills, headache, muscle and joint pain as well as other flu-like symptoms (1, 64).

Since the inception of molecular methods, the detection of these pathogens has been verified in different samples obtained from arthropods and animals, with less frequent occurrence in humans from West Africa (9). While TBRF is endemic in different African countries, outbreaks of LBRF are frequently reported from Ethiopia, Eritrea, Somalia, and South Sudan.

**Figure 6:**
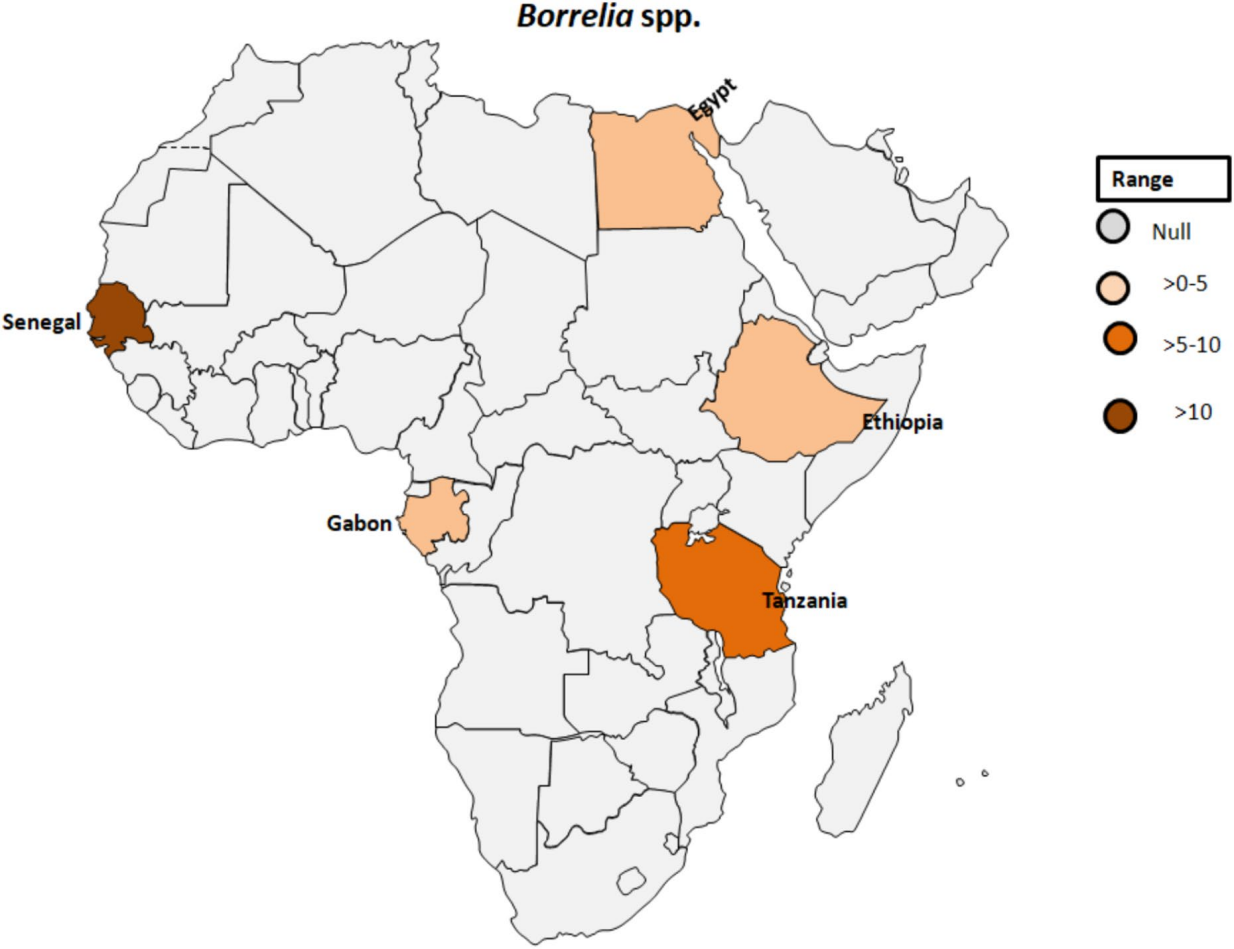
Pooled data using fixed effect model with R version 4.0.1 showing reported Prevalence of *Borrelia* spp. in different African countries designed using Tableau, 2022.2 software.

Regarding the occurrence of TBRF, 115 out of 1,566 samples were tested positive (7.3%) for *B. crocidurae* DNA by Mediannikov et al. (65) in Senegal (see Table 1; Figure 6). In addition, clinical cases of TBRF have been documented, particularly in rural areas in Senegal where individuals may have close contact with ticks (66). In this study, *B. crocidurae* DNA was detected in 7.22% (159/2,202) with a higher prevalence observed during the summer months of July and August.

Grecchi et al. (67) reported a case of relapsing fever caused by *B. recurrentis* in a refugee traveling to Europe from Mali. Two out of five incidences of recurrent fever were reported from refugees originated from Somalia in Turin (Italy) (68). Febrile diseases in rural areas of Africa could be caused by bacteria transmitted by arthropods (14). Thirteen patients tested positive for DNA from these pathogens, including *Borrelia* spp.*, Francisella* group*, Rickettsia bellii, Rickettsia felis,* and *Bartonella rochalimae*. This finding suggests that previously ignored organisms like *Rickettsia*, *Bartonella*, *Francisella*, and *Borrelia* should be considered in empiric therapies, leading to more informed decision-making on the accurate anti-microbial treatment.

A novel *Borrelia* species closely related to *B. crocidurae* or *B. hispanica* was identified in a 24-year-old man with a travel history to Senegal. He presented symptoms including fever, chills, headache, myalgia, arthralgia, and mild diarrhea (69).

In Dielmo, Senegal, a study by Vial et al. (70) reported an average TBRF incidence of 11 cases per 100 person-years over 14 years with variations over time. Another study conducted in Algeria between May 2012 and October 2015 found that 5 out of 48 (10.4%) ticks collected from *Larus michahellis* nests were positive for *Borrelia* spp. Further analysis of *flaB* gene sequences revealed 100% identity with North American genotypes of *B. turicatae* and a 99.77% identity with another *B. turicatae* genotype (71). In a study assessing the prevalence of bacteria and *Plasmodium* spp. in febrile and afebrile children in Franceville, Gabon, *Borrelia* spp. was detected in two controls, while *Rickettsia felis* was found in 10 children (8 febrile, 2 afebrile). No DNA of other microorganisms could be detected in this investigation (72).

In Zambia, *Ornithodoros faini* ticks and bats, particularly *Rousettus aegyptiacus*, were suggested as potential vector and reservoir hosts, respectively, for a *Borrelia* species closely related to New World relapsing fever *borreliae*, raising questions about the evolutionary history and distribution of *Borrelia* species in Southern Africa (73).

Egyptian farmers living in Nile Delta villages exhibited instances of anaplasmosis and TBRF, with evidence of *A. phagocytophilum* infection found in five individuals (7.5%) and DNA of *B. burgdorferi* identified in two samples (3%; Table 1; Figure 6) (27). The relevance of this finding seems to be questionable as Lyme disease *Borrelia* species are exclusively vectored by ixodid ticks known to be absent in Egypt.

### 4.6 Q fever

**Figure 7:**
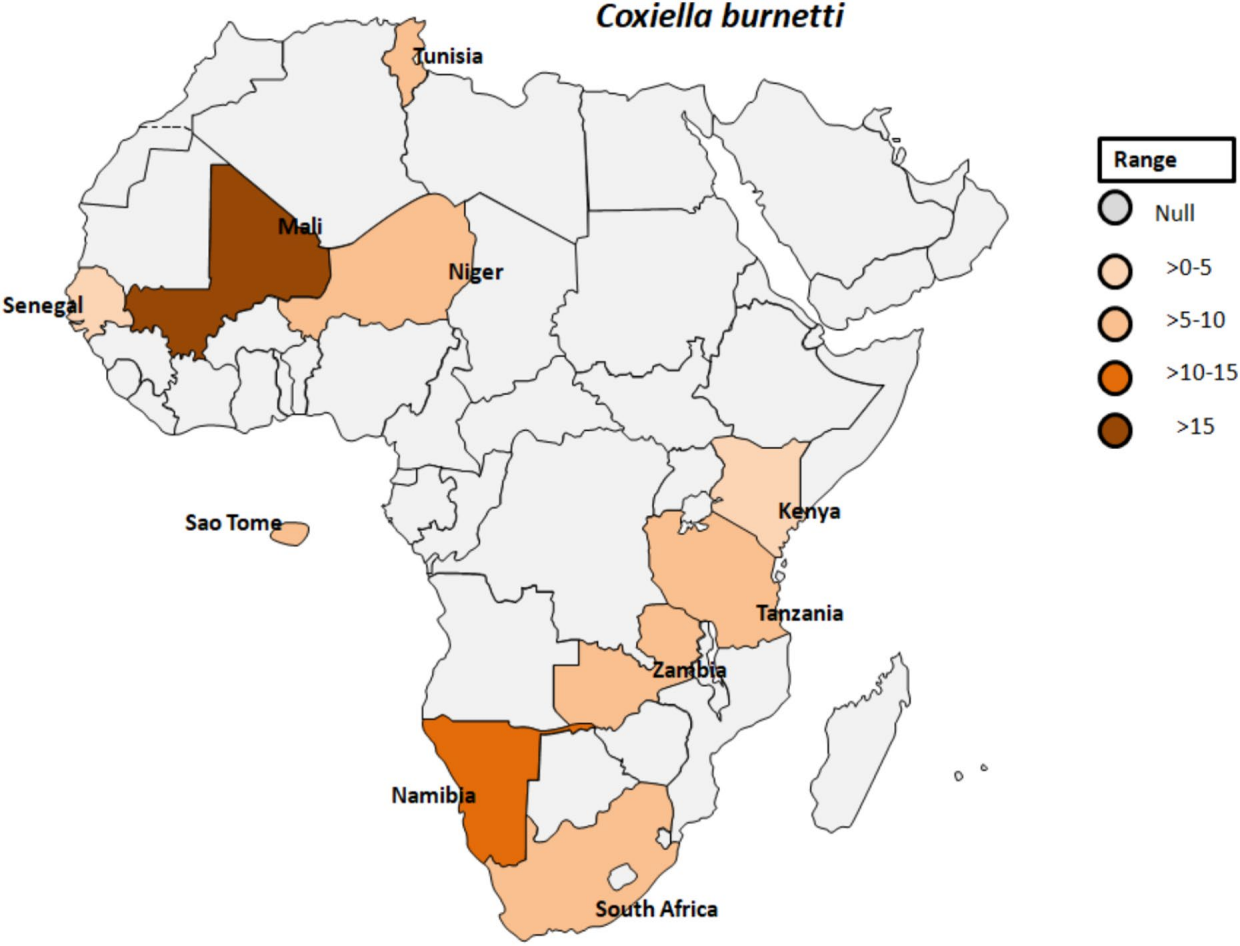
Pooled data using fixed effect model with R version 4.0.1 showing reported Prevalence of *Coxiella burnetti* in different African countries designed using Tableau, 2022.2 software.

*Coxiella burnetii* is an obligate intracellular, Gram-negative bacterium and the causative agent of Q fever. This pathogen is transmitted by various ticks such as *Ornithodorus sonrai, Amblyomma variegatum, Hyalomma* spp., and *Rhipicephalus* spp. across different countries in Africa (Figure 7) (74). The clinical manifestations of Q fever include a febrile illness, pneumonia, and hepatitis, typically appearing 2 to 3 weeks after infection (75). *C. burnetii* naturally infects a range of livestock animals, including cattle, goats, and sheep. The bacteria can be found in various organs of infected animals, such as the placenta, as well as in body fluids like amniotic fluid, urine, and milk (76, 77). Humans can become infected through inhalation of *C. burnetii*-contaminated dust or by consuming unpasteurized milk (78). In Gambia, seropositivity for *C. burnetii* was reported in 3.8 to 9.7% of adult humans and 24.9% of small ruminants in Kiang West, underscoring the interconnectedness of human and animal exposure to *C. burnetii* (Figure 7) (79). Seroprevalence studies have also shown the presence of IgG antibodies to various human pathogenic agents, including *C. burnetii* but also *Rickettsia, Leptospira*, West Nile fever virus, and dengue virus, among peacekeepers deployed to Southern Sudan (80).

### 4.7 Rickettsial infection

The Spotted fever group (SFG) *rickettsiae* comprises a collection of closely related bacteria transmitted to humans primarily through the bite of infected ticks (81, 82). These almost neglected bacteria belong to the genus *Rickettsia* and are associated with a range of febrile illnesses known as spotted fever (83). The distinctive feature of SFG rickettsioses (SFGR) is the development of a characteristic eschar, a necrotic skin lesion at the site of tick attachment (83). While the SFG includes various species, each with unique clinical presentations, they collectively pose significant public health concerns, particularly in regions where the vectors thrive (84, 85). Understanding the diverse species within this group of human pathogenic bacteria is crucial for effective diagnosis, treatment, and control strategies.

**Figure 8:**
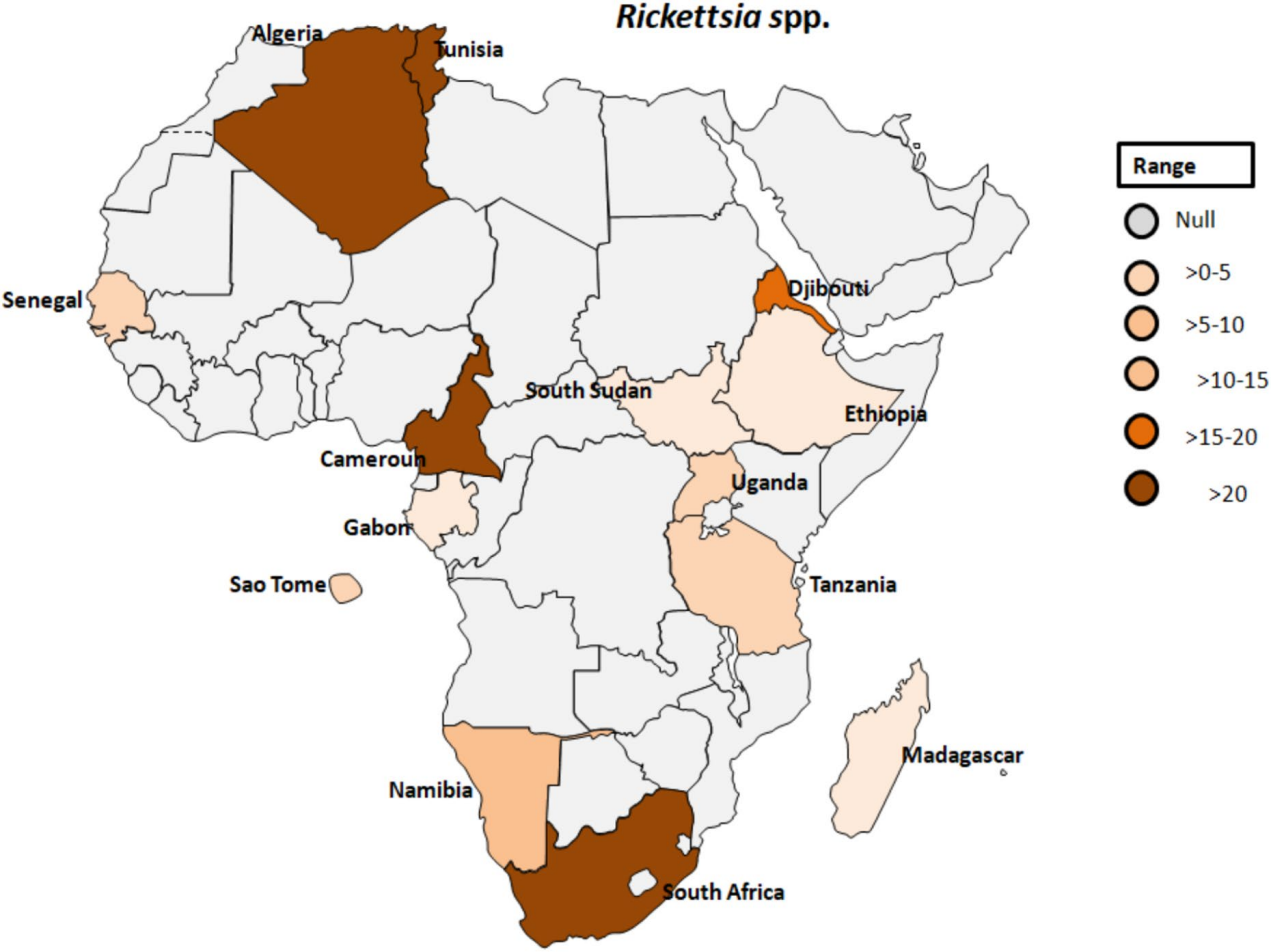
Pooled data using fixed effect model with R version 4.0.1 showing reported Prevalence of *Rickettsia* spp. in different African countries designed using Tableau, 2022.2 software.

*Rickettsia africae*, the causative agent of African tick-bite fever, is transmitted by *Amblyomma* ticks and is endemic in various regions of sub-Saharan Africa (Figure 8). Prior to 2010, Africa reported only one case of Rickettsiosis (86); however, serological evidence of contact with *Rickettsiae* in Dielmo village, Senegal, revealed that 21.4% of 238 individuals tested positive and 51% of 241 ticks from Ndiop village, Senegal, carried *Rickettsia* spp. by molecular identification (87). Additionally, a study in Senegal showed that *Rickettsia* species had a higher prevalence (4.0%) than other bacterial agents in patients with fever of unknown origin, with co-infections of *Plasmodium* spp., Dengue virus, and *Salmonella typhi* (88). In Cameroon, 26.9% of the 903 participants tested positive for antibodies reactive to *R. africae*. However, the observed seroprevalences varied across different regions. Specifically, in Njikwa, the seroprevalence was 51.8%, while in Lomie, it was 38%. In Sobia and Nyabisan, which represent gallery highland and most lowland village sites, the seroprevalences were 37 and 28.7%, respectively (89). This suggests a correlation between the landscapes of the collection sites and the seroprevalence rates among individuals in those regions. In Uganda, rickettsioses and malaria, including Typhus Group Rickettsiosis and SFG *Rickettsia*, were identified as major causes of acute febrile illness in selected clinics (90). Tick collections from domestic and wild animals in Guinea and Liberia, two neighboring countries in tropical West Africa, revealed *Rickettsiae* in nine different tick species. *R. africae* was found in *Amblyomma variegatum* along with other species such as *Rhipicephalus geigyi*, *Rh. annulatus*, *Rh. decoloratus*, and *Amblyomma compressum*. A new *Rickettsia* species, provisionally named Candidatus *Rickettsia liberiensis*, was identified in *Ixodes muniensis* collected from a dog in Liberia (91). Diagnosis of human rickettsiosis typically relies on serological methods (7). However, identification of the causative agents by swabbing eschars in patients with skin lesions after traveling to South Africa (92). Molecular tools targeting distinct genes revealed the presence of *R. africae* in patients who had returned from South Africa, even in cases where serology yielded negative results. A study conducted on 141 adult patients in rural South Africa to determine the prevalence and risk factors of two endemic zoonoses, Q fever and SFGR revealed that 27% of patients were exposed to *Coxiella burnetii* with a higher prevalence among individuals attending cattle inspection facilities (see Table 1; Figure 8) (93). Additionally, 21% of patients had evidence of acute SFGR with higher odds of seropositivity among females and those attending cattle inspection facilities. A study dealing with the prevalence of different zoonoses and the determination of certain risk factors at human-wildlife-livestock interface at Mpumalanga region of South Africa showed that 77% of febrile individuals and 98% of dip-tanksters had at least one positive test for zoonotic pathogens (94). Detection of two fastidious bacteria, *Rickettsia felis* and *Borrelia* spp., in two control samples emphasizes the importance of including controls in the study.

Furthermore, *Rickettsia felis* was identified in 10 children, comprising eight with febrile conditions and two without fever (72). This signifies the importance of including controls for better understanding of the causative agent of fever in sub-Saharan Africa. Unlike *Plasmodium* spp., there has been a lack of investigation into the presence of rickettsial agents in human feces (95). In Ethiopia, 23 out of 102 (22.5%) children with fever were tested positive for one or more zoonotic-transmitted pathogens using real-time PCR. *Rickettsia* spp. were detected in three children and *Borrelia* spp. in two children, of which one child (0.9%) was tested positive for both *Plasmodium* spp. and *Rickettsia* spp. (96). Similarly, SFG *Rickettsiae* were identified in 11.9% of samples tested in Namibia, with male gender as the only significant risk factor (52). There may be a regional trend in exposure, with higher rates in northern regions and the lowest prevalence in the southern region of Hardap in Namibia, with male gender and the 20–29 year age group as the only significant risk factors.

Other studies found regional trends in exposure to spotted fever and typhus group *Rickettsiae,* with sub-acute neuropathy associated with African tick bite fever (ATBF) in Namibia (97). However, this study only represents a selected group of individuals and does not provide a comprehensive prevalence rate for the general population. Therefore, the study by Keller et al. (97) lacks sufficient data to establish the comprehensive prevalence of ATBF and the involvement of neuropathy. Importantly, detailed descriptions of clinical symptoms are lacking. Treatment primarily involved doxycycline for the majority of patients (77%), with other cases receiving thiamphenicol, fluoroquinolones, or macrolide antibiotics. The outcomes were favorable for 8.6% of patients, with the resolution of fever occurring, on average, after 2.92 days. Similarly, rickettsioses were most commonly diagnosed during the summer months of June, July, and August in Namibia (52). In the same study area, severe forms of the disease were observed in 14.7% of patients suffering with neurological manifestations and multi-visceral involvement. However, the study focused on clinical and epidemiological aspects and did not investigate the underlying mechanisms of the disease.

In southwestern Tanzania, the seroprevalence of SFG rickettsiosis was calculated to be 67.9% (Table 1; Figure 8) (98). This study also found that seropositivity was strongly associated with age, gender, higher temperatures during the day, and elevation, with a significant decline above 1,578 meters. Similarly, another study from Algeria revealed increased antibody titres against spotted fever rickettsial antigen in 77% of patients. Although human exposure to infected *Ornithodoros* ticks was observed, evidence of rickettsial DNA in blood samples from villagers was lacking (99). There is a potential link between SPG *Rickettsiae* and unexplained febrile illnesses despite malaria control in Sao Tome (100). The study provided serological evidence in humans for SFG *Rickettsiea* and *C. burnetii*, along with molecular evidence in ticks for SFG *Rickettsiae* in Sao Tome and Principe. In a similar study that focused on the frequency of febrile illnesses caused by vector-borne bacterial pathogens (52), 319 serum samples were collected, with the majority of individuals tested residing in urban settings (81.4%) and regularly interacting with animals (97.3%), including domestic and companion animals. Interaction with cattle, donkeys, and/or horses significantly increased the risk of exposure to *C. burnetii* (52).

Furthermore, in Gabon, zoonotic pathogens previously believed to exclusively infect humans have been found to originate in apes (55). In Djibouti, abattoir workers exhibited seropositivity for SFG *Rickettsiae*, typhus group *Rickettsiae*, and *Orientia* spp. This marks the first evidence of exposure to this *Rickettsia* spp. in the Horn of Africa (101). *Orientia* species are responsible for scrub typhus, which is closely related to the clinical symptoms of rickettsioses (102), but mainly found in Asia and Australia (57). Similarly, serological evidence of rickettsial infection was confirmed in 57.5%, and Q-fever diagnosed in 8.5% in Tunisia (103). Despite the significant annual cases of rickettsial infection recorded in Tunisia, the causative agent(s) have, regrettably, not been conclusively identified (104). However, a study employing quantitative real-time PCR and a Reverse Line Blot test did identify the presence of rickettsial DNA in skin biopsies and swabs, with *Rickettsia conorii* being the most prevalent bacterial species (104).

## 5 Challenges in distinguishing between different etiological agents of fever of unknown origin

Access to reliable diagnostic testing in most African countries is limited, and traditional diagnostic methods face challenges (11), prompting the establishment of a Point-of-Care (POC) laboratory (105). The POC laboratory was designed to address specific challenges in rural settings. Molecular-based POC testing utilizing real-time PCR was implemented for diagnosing various infectious diseases (105). Recently, mass spectrometry was established as an alternative method for species identification, but this diagnostic tool is highly cost-intensive and requires well-trained personnel (95). Distinguishing between different etiological agents of tick-borne febrile illnesses poses significant challenges due to overlapping clinical presentations and diagnostic limitations:

- Non-specific Symptoms: Many tick-borne diseases share common clinical symptoms, such as fever, fatigue, myalgia, and arthralgia, making it difficult to clearly differentiate between each disease solely on clinical parameters. Common clinical manifestations among patients with positive PCR results for *Rickettsiae* included headaches (100%), chills (93.8%), muscle aches (68.8%), joint pains (68.8%), and rash (4.4%) (106).
- Limited access to diagnostics: In many resource-limited settings in Africa, access to advanced diagnostic tests is restricted, leading to reliance on clinical diagnosis or basic laboratory tests that may lack specificity. The disease was initially screened at the dispensary, followed by a second screening in Dakar conducted by highly trained personnel. Among the samples that tested positive, only 4 (15%) were identified as positive by thick smears at the dispensary, while 15 (56%) were confirmed positive during the second screening (107). In Senegal, MALDI-TOF MS was shown to be a valuable tool for tick species identification in the study of ticks, enabling species identification, detection of tick-associated microorganisms, and comparison of preservation methods (13). It provides researchers with important insights into tick biology and tick-borne diseases. There is the emergence of new approaches, like swabbing eschars for PCR testing (77). Rickettsial diagnosis is challenging in routine laboratories, and serology provides only a retrospective diagnosis (28). Thus, molecular methods, particularly qPCR, are proposed for routine diagnosis to overcome the obvious limitations of antibody testing (63). However, despite the advantage of the implementation of PCR-based methodologies in routine diagnosis, a cost-effective evaluation, and cost-intensive equipment, as well as well-trained technicians are required, which is often nearly impossible to realize in rural settings (63). Recently, Röttgerding et al. [12] developed two novel immunoassays (line immunoblot and ELISA) for IgM and IgG, employing complement-inhibiting protein (CihC) and glycerophosphodiester phosphodiesterase (GlpQ) of *B. recurrentis* as promising candidates for the diagnosis of louse-borne relapsing fever.
- Co-infections: Co-infections with multiple tick-borne pathogens can further complicate diagnosis and treatment decisions, as symptoms may be masked or exacerbated. Liu et al. (106) suggested the inclusion of rickettsial infections in the comprehensive diagnostic evaluation of febrile cases within region in western Kenya. Furthermore, there is a distinct recommendation for the establishment of diagnostic capabilities tailored specifically to rickettsial infections, particularly in locales characterized by a high prevalence of malaria.
- Cross-reactivity: Cross-reactivity refers to the ability of antibodies produced in response to the infection of a specific pathogen to react with similar antigens from other pathogens (108). In the context of tick-borne febrile illnesses, this phenomenon can complicate diagnostic efforts, thus leading to potential misidentifications and, consequently, misguided treatment strategies (109). The cross-reactivity issue is particularly pronounced in serological tests, where antibodies may not distinguish between different pathogens, causing false-positive or false-negative results (110). Several factors contribute to cross-reactivity in tick-borne febrile illnesses (111). Furthermore, the coexistence of multiple pathogens within the same geographic area, coupled with overlapping tick vectors, enhances the likelihood of cross-reactivity (112).

Cross-reactivity in tick-borne febrile illnesses has profound implications, affecting the accuracy of *in vitro* diagnostic and subsequent treatment decisions (Table 2) (113). The challenges posed by cross-reactivity highlight the pressing need for more specific and targeted diagnostic tools to differentiate closely related pathogens (76). False-positive results may lead to unnecessary treatments, while false negatives can result in delayed or inadequate interventions.

**Table 2:** Available diagnostics, diagnostic limitations, and expected improvements for bacterial tick-borne infections in Africa.

Pathogen	Available diagnostics	Diagnostic limitations	Expected improvements
*Anaplasma* spp.	Serology (IFA, ELISA), PCR	Cross-reactivity with other pathogens, low sensitivity in early infection	Development of more specific and sensitive PCR assays, point-of-care testing kits
*Ehrlichia* spp.	Serology (IFA, ELISA), PCR	Similar issues as with *Anaplasma*, limited availability of PCR in endemic areas	Improved molecular diagnostics, wider availability of PCR, rapid diagnostic tests
*Rickettsia* spp.	Serology (IFA, ELISA), PCR	Serology can be nonspecific, PCR not widely available	Enhanced PCR methods, multiplex assays to differentiate *Rickettsia* species
*Borrelia* spp.	Serology (ELISA, Western Blot), PCR, Culture	Serology may miss early/late cases, culture is slow and complex	Next-generation sequencing for comprehensive detection, improved serological assays
*Coxiella burnetii*	Serology (IFA, ELISA), PCR	Chronic Q fever diagnosis is challenging, PCR not always available	Better chronic infection markers, increased PCR availability
*Bartonella* spp.	Serology (IFA, ELISA), PCR, Culture	Difficult to culture, serology shows cross reactivity, PCR sensitivity varies	Advanced culture techniques, species-specific PCR, improved serological tests
*Francisella tularensis*	Serology (agglutination, ELISA), PCR, Culture	High biosafety level required for culture, serology shows cross-reactivity	Safer, more sensitive molecular diagnostics, rapid antigen detection tests

IFA, indirect fluorescent antibody.

Currently, *in vitro* diagnostics employing immunological and molecular methods, such as ELISA, line blots, or various PCR-based assays, may encounter challenges in mitigating cross-reactivity. Although mass spectrometry and point-of-care testing show promise, obstacles persist in their widespread adoption, particularly in resource-limited settings where many tick-borne diseases are prevalent (77). In conclusion, to address the challenges known in the diagnosis of tick-borne diseases, research efforts should focus on developing more specific and reliable diagnostic tools. Targeting unique antigens associated with each pathogen, exploring the potential of next-generation sequencing technologies, and implementing advanced techniques like mass spectrometry offer new opportunities for improving accuracy in species identification and differentiation (77).

## 6 Conclusion

In conclusion, this review has delved into the complicated nature of tick-borne febrile illnesses in humans in Africa. Tick-borne febrile illnesses are undeniably significant health concerns in Africa, given the multiple human pathogens transmitted by ticks, including *Anaplasma* spp.*, Bartonella* spp.*, Borrelia* spp.*, Coxiella burnetii, Ehrlichia* spp.*, Francisella tularensis*, and *Rickettsia africae* (1, 60, 61, 63, 114). The epidemiological pattern of these diseases is linked with tick habitats, prevalent in rural and semi-urban areas where humans and livestock closely interact with tick habitats (8, 52). Despite the significant impact of tick-borne diseases on the well-being of economically disadvantaged farming communities in developing countries, diagnostic limitations pose substantial challenges (63). The overlapping clinical presentations, non-specific symptoms, and limited access to current diagnostic tests in resource-limited settings contribute to the complexity of achieving accurate diagnoses.

The exploration of aetiologies, through a thorough literature search from January 1990 to June 2024, has revealed the diversity of tick-borne pathogens affecting human populations in Africa. By acknowledging the diagnostic challenges and emphasizing the urgency for improvement, this review contributes to the broader understanding of tick-borne febrile illnesses, paving the way for advancements in diagnostics that are crucial for disease prevention and treatment in the African context.

The exploration of the unique features of hard ticks (*Ixodidae*) and soft ticks (*Argasidae*) further enriches the understanding of the complex life cycles and transmission dynamics of these vectors highlighting the importance of addressing transovarial transmission for effective disease control. In essence, this review serves as a valuable resource for health professionals, researchers, and policymakers urging collaborative efforts to enhance diagnostic capabilities, promote awareness, develop novel *in vitro* diagnostics (simple but specific POC tests for low-income countries, preferred created as a multiplex test), and strategies for mitigating the impact of tick-borne febrile illnesses on public health in Africa.

## Appendix 1

List of articles included in the review.

*Anaplasma phagocytophilum*: (27, 28, 115, 116), *Bartonella* spp.: (14, 50, 51, 94, 103, 105), *Borrelia* spp.: (14, 27, 70, 72, 96, 105, 107, 117–122), *Coxiella burnetii*: (52, 79, 80, 93, 94, 100, 103, 105, 123–129), *Ehrlichia* spp.: (35), *Francisella* spp.: (14, 130), *Rickettsia* spp.: (14, 52, 72, 80, 87–90, 93, 94, 96–101, 103, 104, 117, 120, 131–135).
